# Lung Injury Is a Predictor of Cerebral Hypoxia and Mortality in Traumatic Brain Injury

**DOI:** 10.3389/fneur.2020.00771

**Published:** 2020-08-07

**Authors:** Chiara Robba, Shadnaz Asgari, Amit Gupta, Rafael Badenes, Mypinder Sekhon, Erta Bequiri, Peter J. Hutchinson, Paolo Pelosi, Arun Gupta

**Affiliations:** ^1^Anaesthesia and Intensive Care, San Martino Policlinico Hospital, IRCCS for Oncology and Neuroscience, Genoa, Italy; ^2^Biomedical Engineering Department, California State University, Long Beach, CA, United States; ^3^Computer Engineering and Computer Science Department, California State University, Long Beach, CA, United States; ^4^Emergency Department, Broomfield Hospital, Mid-Essex Hospital Trust, Essex, United Kingdom; ^5^Department of Surgery, University of Valencia, Valencia, Spain; ^6^Division of Critical Care Medicine, Department of Medicine, Vancouver General Hospital, University of British Columbia, Vancouver, BC, Canada; ^7^Department of Neurosurgery, Addenbrooke's Hospital, Hills University of Cambridge, Cambridge, United Kingdom; ^8^Department of Physiology and Transplantation, Milan University, Milan, Italy; ^9^Dipartimento di Scienze Chirurgiche e Diagnostiche Integrate, Università Degli Studi di Genova, Genoa, Italy; ^10^Neurocritical Care Unit, Addenbrooke's Hospital, Cambridge, United Kingdom

**Keywords:** mortality outcome, traumatic brain injury, cerebral oxygenation, partial arterial oxygen pressure, lung injury, hypoxia threshold

## Abstract

**Background:** A major contributor to unfavorable outcome after traumatic brain injury (TBI) is secondary brain injury. Low brain tissue oxygen tension (PbtO2) has shown to be an independent predictor of unfavorable outcome. Although PbtO2 provides clinicians with an understanding of the ischemic and non-ischemic derangements of brain physiology, its value does not take into consideration systemic oxygenation that can influence patients' outcomes. This study analyses brain and systemic oxygenation and a number of related indices in TBI patients: PbtO2, partial arterial oxygenation pressure (PaO2), PbtO2/PaO2, ratio of PbtO2 to fraction of inspired oxygen (FiO2), and PaO2/FiO2. The primary aim of this study was to identify independent risk factors for cerebral hypoxia. Secondary goal was to determine whether any of these indices are predictors of mortality outcome in TBI patients.

**Materials and Methods:** A single-centre retrospective cohort study of 70 TBI patients admitted to the Neurocritical Care Unit (NCCU) at Cambridge University Hospital in 2014–2018 and undergoing advanced neuromonitoring including invasive PbtO2 was conducted. Three hundred and three simultaneous measurements of PbtO2, PaO2, PbtO2/PaO2, PbtO2/FiO2, PaO2/FiO2 were collected and mortality at discharge from NCCU was considered as outcome. Generalized estimating equations were used to analyse the longitudinal data.

**Results:** Our results showed PbtO2 of 28 mmHg as threshold to define cerebral hypoxia. PaO2/FiO2 found to be a strong and independent risk factor for cerebral hypoxia when adjusting for confounding factor of intracranial pressure (ICP) with adjusted odds ratio of 1.78, 95% confidence interval of (1.10–2.87) and *p*-value = 0.019. With respect to TBI outcome, compromised values of PbtO2, PbtO2/PaO2, PbtO2/FiO2, and PaO2/FiO2 were all independent predictors of mortality while considered individually and adjusting for confounding factors of ICP, age, gender, and cerebral perfusion pressure (CPP). However, when considering all the compromised values together, only PaO2/FiO2 became an independent predictor of mortality with adjusted odds ratio of 3.47 (1.20–10.04) and *p*-value = 0.022.

**Conclusions:** Brain and Lung interaction in TBI patients is a complex interrelationship. PaO2/FiO2 seems to be a major determinant of cerebral hypoxia and mortality. These results confirm the importance of employing ventilator strategies to prevent cerebral hypoxia and improve the outcome in TBI patients.

## Introduction

Traumatic brain injury (TBI) is a leading cause of death and disability (1). A characteristic feature of TBI is a wide variation in functional outcome (2). Clinicians treating patients with TBI often make therapeutic decisions based on the assessment of prognosis (3). Several studies have attempted to find parameters that allow the clinicians to assess the risks and outcomes of the patient after TBI. These include clinical and demographic variables such as age, gender, race, Glasgow Coma Score (GCS), which includes motor score and pupil reactivity, and cause of injury (4).

A major contributor to unfavorable outcomes is secondary brain damage which progresses hours or days after TBI (5). It is widely accepted that causes of secondary injury include impaired cerebral metabolism, hypoxia, and ischemia (5). Currently, severe TBI care is centered on control of intracranial pressure (ICP) and cerebral perfusion pressure (CPP), where invasive ICP monitoring is the gold standard monitor (6). However, studies have shown that cerebral hypoxia after severe TBI can occur despite ICP and CPP being within normal ranges, and this note should be taken into consideration when making a decision to enhance treatment for ICP management (5–7). Furthermore, cerebral hypoxia causing cellular metabolic dysfunction may precede a rise in ICP, implying diffusion abnormalities rather than abnormalities with perfusion (5).

Several studies suggested that cerebral hypoxia is also an independent factor associated with unfavorable outcome (7–9). Subsequently, brain tissue oxygen tension (PbtO2) and microdialysis have been introduced in some institutions as advanced brain monitoring methods to complement ICP monitoring.

Although PbtO2 provides clinicians with an understanding of the ischemic and non-ischemic derangements of brain physiology (9, 10), there are some unanswered questions. Firstly, it is not exactly known which parameters are the major determinants of PbtO2. Hemoglobin, cerebral perfusion pressure, and systemic oxygenation seem to have an important role (10, 11); however, it is not clear which specific variable has the predominant role in the occurrence of cerebral hypoxia. Secondly, uncertainty exists regarding the critical threshold that defines poor outcome and whether monitoring of PbtO2 influence the treatment and hence the outcome (12).

The most recent brain trauma foundation guidelines suggest that “hypoxia detected by monitors is associated with worse outcomes” (13). However, PbtO2 value alone does not take into consideration systemic and ventilator parameters such as partial arterial oxygen tension (PaO2), which are often crucial in the neurocritical care population (14). We therefore conducted a retrospective study, with the primary aim of indicating the threshold of cerebral hypoxia and parameters that are determinants of cerebral hypoxia. Our secondary goal was to assess whether any of these indices (PbtO2, partial arterial oxygenation pressure (PaO2), PbtO2/PaO2, ratio of PbtO2 to fraction of inspired oxygen (FiO2), and PaO2/FiO2) based on the relationship between cerebral tissue oxygenation and systemic arterial oxygenation and the fraction of inspired oxygen, can be useful for prognostication of mortality in TBI patients.

## Materials and Methods

This is a single-centre retrospective cohort study. The study was approved by the relevant research ethic committee (30REC97/291) for anonymized data recording.

### Patient Inclusion, Data Collection, and Management

Patients were identified using the EPIC system^®^ which contains a database of all patients admitted to NCCU at Cambridge University Hospitals between November 2014 and October 2018. The patients included in this study were adult TBI requiring advanced neuromonitoring with invasive ICP and PbtO2 captured using ICM +^®^ brain monitoring software (Cambridge Enterprise Ltd, Cambridge, UK; http://www.neurosurg.cam.ac.uk/icmplus). ICP was monitored with an intraparenchymal sensor (Codman ICP Micro-Sensor; Codman & Shurtleff, Raynham, MA). Licox sensors (Codman Raynham, MA) were inserted through a cranial access device (Technicam, Newton Abbot, UK). The probe was inserted in the perilesional areas. Arterial blood pressure (ABP) was zeroed at the level of the middle cranial fossa (Baxter Healthcare Health Care Corp. Cardio-Vascular Group, Irvine, CA). Arterial blood gases were obtained through an invasive catheter in the radial artery and concomitant PaO2 and PbtO2 values were recorded along with fraction of inspired oxygen. The collected data was saved into a standardized and secure electronic spreadsheet using Microsoft Excel 2013^®^ (Redmond, WA, USA). The following characteristics were manually collected and added to the dataset: demographics of the patients, hemoglobin level (Hb), Glasgow Coma Scale (GCS) at admission and mortality at discharge from NCCU.

Patients were managed according to current TBI guidelines and management protocol (15), based on a staircase approach with increasing level of therapy including intravenous sedation, neuromuscular paralysis, therapeutic hypothermia, hyperosmolar therapy, cerebral spinal fluid diversion using external ventricular drains, and surgical decompression. In general, management endpoints include maintaining CPP >60 mmHg and PbO2>20 mmHg in our unit (13, 15–17). No withdrawal of life sustaining therapies was applied in the study population.

### Statistical Analysis

Our dataset consisted of instantaneous values of several variables (ICP, CPP, Hb, FiO2) at time instances when measured values for both PbtO2 and PaO2 were available. Due to repeated simultaneous measurements of variables at different time points and for different subjects (longitudinal data), generalized estimating equations (an extension of generalized linear models) with a covariance matrix structured by an autoregressive model were employed (11). A *p*-value smaller than 0.05 was considered as statistically significant. All the analyses were conducted in Matlab R2017b (Mathworks, Natick, MA). Throughout this paper, the term “episode” refers to each simultaneous measurement of all variables. If PbtO2<Threshold for an episode, then that episode is referred as “an episode with compromised PbtO2” or “hypoxic.”

#### PbtO2 Threshold for Cerebral Hypoxia

In our study, we conducted two analyses to further investigate the PbtO2 threshold: (A) We calculated the correlation of the percentage of hypoxic episodes (defined as Pbto2<Threshold) and the mortality outcome when Threshold value was changed from 7 to 40 mmHg. (B) We also obtained the odds ratio (OR) and its 95% confidence interval (CI) for mortality detection by applying generalized estimating equations when the compromised PbtO2 (dichotomized PbtO2<Threshold or PbtO2≥Threshold) were entered to the model as input, mortality outcome was considered as the response variable while adjusting for confounding factors such as ICP, CPP, age and gender by considering them as covariates (11).

#### Determinant of Cerebral Hypoxia

Our goal was to determine whether PaO2/FiO2 can be used as a surrogate of PbtO2 and consequently as a predictor of hypoxia. For this purpose, PbtO2 was considered as the response variable. PaO2/FiO2 was entered into the model as continuous input variable while adjusting for confounding factors such as PaO2, CPP, and Hb by considering them as covariates in the model. To find an optimal threshold on PaO2/FiO2 value to indicate cerebral hypoxia, we plotted the mean and 95% CI of PaO2/FiO2 values over dichotomized group of episodes: those with compromised PbtO2 (PbtO2<Threshold), and those with normal PbtO2 values (PbtO2≥Threshold) when Threshold was changed over a range of 10–32 mmHg. We also calculated the probability of the two-sample *t*-test that the difference between average of compromised and normal PbtO2 episodes is significant.

#### Predictors of Mortality

We aimed to identify those variables (among PaO2, PbtO2, PbtO2/PaO2, PbtO2/FiO2, PaO2/FiO2) that were strong and independent predictors of mortality. For this purpose, we found the threshold value for each variable that can distinguish death from survival outcomes by calculating the percentage of episodes where the variable was smaller than the threshold value for each patient. The mean and 95% CI of these percentages were obtained and compared between patients who died and those who survived. Then the most predictive (optimal) threshold of mortality was identified as the threshold value where the difference between the death and survival plots are maximized in a statistically significant manner (when probability of *t*-test is smaller than 0.05). Each variable was then dichotomized using the obtained threshold value (variable< Threshold vs. variable ≥ Threshold) and entered into the model as input whereas the mortality outcome was considered as the response variable. The association of each dichotomized variable and mortality was also adjusted for confounding factors ICP, CPP, age, and gender.

## Results

During the period of study, a total of 303 measurements from 70 patients fulfilled the inclusion criteria outlined previously. Table 1 summarizes the baseline data of the study cohort. The mean age of the cohort was 43 ± 20. The average number of repeated measurements per subject was 4.

**Table 1:** Baseline data of study cohort.

	**Dead**	**Survived**	***P*-value**
*N*	13	57	
Age (years)	63 ± 17	39 ± 18	<0.001^*^
Male (%)	76.9	70.2	0.608
GCS	4 (3–7.5)	14 (10.5–15)	<0.001^*^
PaO2	112.0 ± 36.9	116.8 ± 30.3	0.328
FiO2	0.5 ± 0.2	0.4 ± 0.1	<0.001^*^
Pbto2	18.8 ± 8.8	26.0 ± 11.3	<0.001^*^
Pbto2/PaO2	0.18 ± 0.09	0.23 ± 0.11	<0.001^*^
Pbto2/FiO2	42.2 ± 25.2	75.6 ± 42.2	<0.001^*^
PaO2/Fio2	242.3 ± 85.2	326.0 ± 99.0	<0.001^*^
ICP	14.6 ± 7.9	9.6 ± 5.0	<0.001^*^
CPP	72.4 ± 12.7	76.5 ± 8.2	0.004^*^
Hb	9.56 ± 0.24	9.63 ± 0.11	0.002^*^
Number of measurements per patient	3 (1–6)	4 (2.75–6)	0.535

*Numerical data are expressed as mean ± SD and compared with one-way ANOVA with repeated measurements. Categorical data are expressed as number (percentage) or median (IQR) and compared with chi-square test. N, number; GCS, Glasgow Coma Scale; PaO2, partial pressure of oxygen; FiO2, inspired fraction of oxygen; PbtO2, brain tissue oxygen tension; ICP, intracranial pressure; CPP, cerebral perfusion pressure; Hb, Hemoglobin*.

* *Statistically significant*.

Median admission GCS was 12.5 (6–15). Median number of measurements per patient was 4 (2–6) and not significantly different between those who survived and died. All other variables except gender and PaO2 values were significantly different between the two outcomes.

### PbtO2 Threshold for Cerebral Hypoxia

**Figure 1:**
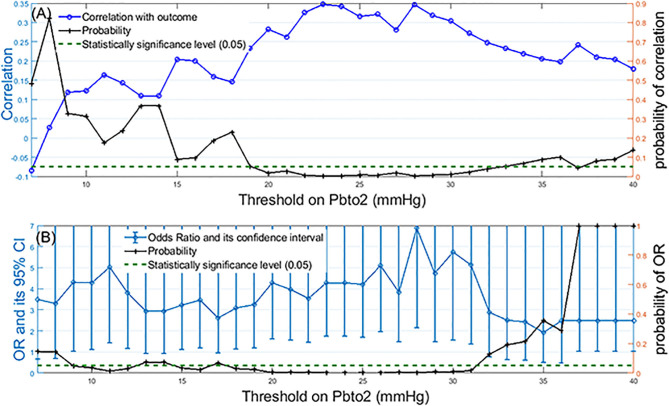
Identification of the optimal threshold on PbtO2 to define hypoxia: **(A)** Left-side vertical axis shows correlation values between the percentage of episodes with compromised PbtO2 and the mortality outcome over all patients when the PbtO2 threshold (shown as horizontal axis) changed from 7 to 40 mmHg. Right-side vertical axis displays the probability of the significance of the calculated correlation for each threshold value. **(B)** Left-side vertical axis displays odds ratio and its 95% confidence interval for mortality detection by using dichotomized PbtO2 (PbtO2<Threshold or≥Threshold) as the input of generalized estimating equations while adjusting for confounding factors such as intracranial pressure (ICP), cerebral perfusion pressure (CPP), age and gender. The right-side vertical axis displays the probability associated with calculated OR. The dashed green line represents the probability significance level of 0.05. So, any probability below the dashed green line is statistically significant.

Figure 1A presents the correlation plot along with probability of its significance (right vertical axis) for when threshold value changes from 7 to 40 mmHg. We observe that the statistically significant correlation (*p*-value < 0.05) becomes more than 0.25 when hypoxic threshold is defined in the range of 20–31 mmHg. Figure 1B presents Odds Ratio (OR) and its 95% Confidence Interval (CI) for mortality detection along with its statistical significance (probability on the right vertical axis). We observe that the statistically significant odds ratio becomes equal or more than 4 when PbtO2 threshold (and therefore to define hypoxia) is in the range of 20–31 mmHg.

**Figure 2:**
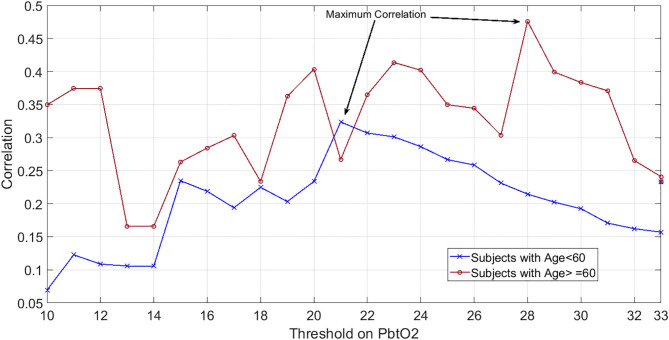
Showcasing dependency of hypoxic PbtO2 threshold to subjects' age: Correlation values between the percentage of episodes with compromised PbtO2 and the mortality outcome over patients when PbtO2 threshold (shown as horizontal axis) changed from 7 to 40 mmHg. Blue plot indicates the patients younger than 60 years old while red plot shows the correlation results for those who are 60 years old or older.

In our dataset both correlation and OR were maximized at the threshold of 28 mmHg. Further grouping of the patients into two age categories (those with age of ≥60 years old vs. those younger than 60 years old) revealed that although the maximum correlation for older subjects occurs at PbtO2 threshold of 28, this threshold value decreases to 20 mmHg for the younger patients (Figure 2).

From a total of 303 episodes of measurements, 186 episodes had Pbto2<28. Among these, only 12 had ICP>20 and 6 episodes had CPP<60. Thus, prevalence of ICP elevation among hypoxic episodes (12/186 or 6.1%) was comparable with general prevalence of ICP elevation over all episodes (18/303 or 5.9%) (9). Also, 60.6% of the hypoxic episodes (Pbto2 ≤ 28) had ICP value lower than 20 and CPP value more than 60 mmHg.

### Determinants of Cerebral Hypoxia

CPP and PbtO2 had a non-statistically significant correlation of 0.1 (*p* = 0.063) considering the overall population. Hb and PbtO2 had a statistically non-significant correlation of 0.14 (*p* = 0.084). PaO2/FiO2 revealed a statistically significant correlation of 0.22 (*p* < 0.001) with brain tissue oxygenation. The results of generalized estimating equations revealed an independent and strong linear correlation between brain oxygenation and PaO2/FiO2 (adjusted *p* < 0.001).

**Figure 3:**
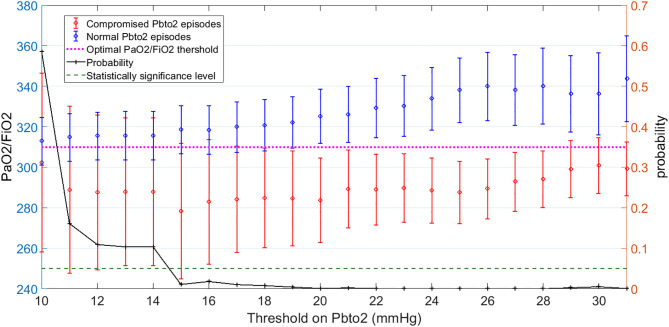
Left-side vertical axis shows the average and 95% Confidence Interval (CI) of PaO2/FiO2 values for two groups of episodes: Those with compromised PbtO2 values of PbtO2<Threshold (in red) and those with normal PbtO2 values of PbtO2≥Threshold (in blue). Right-side vertical axis indicates the probability of the *t*-test that the average of PaO2/FiO2 values are significantly different between the normal (blue) and compromised episodes (red). The vertical axis displays the Threshold values on PbtO2. The dashed green line represents the probability significance level of 0.05. So, whenever probability of the *t*-test (in black) is below the dashed green line, PaO2/FiO2 values are significantly different between those episodes with compromised and normal PbtO2 values: This happened when PbtO2 Threshold is within the range of 20–30 mmHg. The average value of PaO2/FiO2 for when PbtO2 Threshold is within the range of 20–30 corresponds to 310 (The dashed pink line). Thus, PaO2/FiO2 = 310 can be employed as the optimal threshold value on PaO2/FiO2 to define a hypoxic episode.

Figure 3 shows the mean and 95% CI of PaO2/FiO2 over dichotomized group of episodes based on PbtO2 threshold. The right axis displays probability of two-sample *t*-test that the difference between average of compromised and normal PbtO2 episodes is significant. We observe that if the hypoxia threshold is defined in the acceptable range of 20–30 mmHg as discussed before, then a PaO2/FiO2 in the range of 300–320 can result in reasonable separation between compromised and normal Pbto2 episodes and consequently in identifying cerebral hypoxia. For simplicity, in this work, we choose the middle of the range as the optimal threshold of PaO2/FiO2 (PaO2/FiO2<310) to indicate hypoxia for our dataset. Finally, analyzing independent association between dichotomized PaO2/FiO2 (PaO2/FiO2< or ≥ 310) and hypoxia (defined as PbtO2 < 28) results revealed that in fact, PaO2/FiO2 < 310 is an independent risk factor for compromised PbtO2 in our dataset with adjusted odds ratio of 1.78, 95% confidence interval of (1.10–2.87) and *p*-value = 0.019 (Figure 3).

### Predictors of Mortality

All variables except PaO2 showed statistically significant correlation with mortality: PbtO2 (correlation = −0.24, *p*-value < 0.001), PbtO2/PaO2 (correlation = −0.20, *p*-value < 0.001), PbtO2/FiO2 (correlation = −0.30, *p*-value < 0.001), PaO2/FiO2 (correlation = −0.31, *p*-value < 0.001). Correlation value of PaO2 with outcome was non-significant (correlation = −0.06, *p*-value = 0.328).

**Figure 4:**
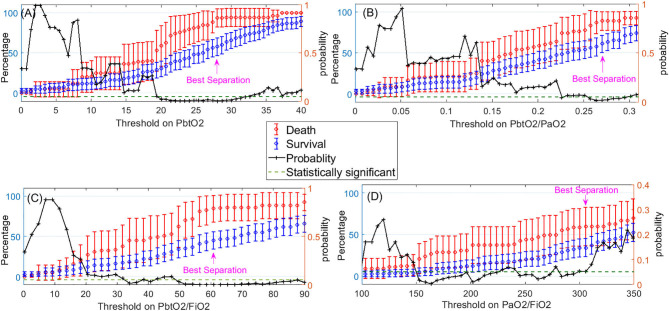
Left-side vertical axis shows the average and 95% confidence interval for the percentages of compromised episodes of a variable (when the variable<Threshold) for two groups of patients: those who died (in red) and those who survived (in blue). The right-side vertical axis indicates the probability of the *t*-test that percentage of compromised episodes is significantly different between those who died and survived. The dashed green line represents the probability significance level of 0.05. So, whenever probability of the *t*-test (in black) is below the dashed green line, difference between death and survival is statistically significant. The pink arrow displays where the best separation between the death and survival outcomes is achieved. This point can be used to obtain the optimal threshold value on each of the following variables to indicate a hypoxic episode: **(A)** PbtO2, **(B)** PbtO2/PaO2, **(C)** PbtO2/FiO2, **(D)** PaO2/FiO2.

Figure 4 displays the mean and 95% CI of the percentages of compromised episodes for both death and survival outcomes. Using these plots, for each variable, we identified the most predictive (optimal) threshold of mortality as the threshold value where the difference or separation between the death and survival plots are maximized in a statistically significant manner. The most predictive threshold on Pbto2, Pbto2/Pao2, Pbto2/Fio2, and PaO2/FiO2 are 28, 0.27, 60, and 310, respectively.

Table 2 presents the adjusted odds ratios, its 95% CI and *p*-values of death detection using the dichotomized version of each variable by applying the optimal threshold we just obtained. We observe that PbtO2<28, PbtO2/PaO2<0.27, PbtO2/FiO2<60, and PaO2/FiO2<310 are each independent predictor of mortality when considered individually.

**Table 2:** Odds Ratio (95% confidence interval) and *p*-value of death detection using the dichotomized version of each variable (variable< Optimal Threshold).

**Compromised value of the variable**	**Odds ratio (95% CI)**	***p*-value**
PbtO2<28	6.88 (2.13–22.23)	0.001^*^
PbtO2/PaO2<0.27	6.73 (2.14–21.14)	0.001^*^
PbtO2/FiO2<60	10.20 (3.64–28.61)	<0.001^*^
PaO2/FiO2<310	4.54 (1.83–11.29)	0.001^*^

* *Statistically significant*.

Finally, entering all these dichotomized variables as the inputs of the model and mortality outcome as the output while adjusting for confounding factors revealed that while each of these dichotomized variables were individually an independent predictor of mortality, when one considered all compromised values together, then PaO2/FiO2 became the independent predictor of mortality with adjusted odds ratio of 3.47 (1.20–10.04) and *p*-value = 0.022.

## Discussion

Our results demonstrated that: (1) Cerebral hypoxic threshold ranges from 20 to 31 mmHg; the optimal hypoxic threshold for patients older than or equal to 60 years old is 28 mmHg, while this threshold decreases to 21 mmHg for those younger. (2) PaO2/FiO2 is significantly correlated with PbtO2, whereas CPP and Hb have a statistically non-significant correlation with PbtO2. PaO2/FiO2<310 is an independent risk factor for cerebral hypoxia. (3) PbtO2, PbtO2/PaO2, PbtO2/FiO2, and PaO2/FiO2 are each independent predictor of mortality. When considering all these compromised values together PaO2/FiO2 becomes the most independent predictor of morality.

Pathophysiology of brain tissue oxygenation is complicated and dependent on several factors including oxygen delivery (cerebral blood flow), arterial oxygen content, oxygen diffusion from the capillary into the mitochondria and mitochondrial oxygen consumption. Brain tissue oxygen is a good clinical marker to identify ischemic and non-ischemic derangements of brain physiology (12). Some studies showed that TBI patients who did not survive, had lower PbtO2 values than those who did survive (11, 12, 18, 19).

Although many have suggested PbtO2 of 20 mmHg as a threshold associated with unfavorable outcome in brain injured patients (8, 18), the optimal threshold of PbtO2 that correlates with bad outcome is not clear and widely varies between studies (20, 21). Meixensberg et al. (20) found no statistical improvement in outcome at 6 months, when PbtO2 was targeted above 10 mmHg. In a prospective study including TBI patients (22), patients treated with ICP and brain tissue PbtO2 monitoring were compared with controls who had undergone ICP monitoring alone, using a PbtO2 target of 25 mmHg. The authors found a significantly reduced mortality rate (44 vs. 25%, *p* < 0.05) in the group monitored with PbtO2. The recently published BOOST-II study (23)-a randomized controlled trial comparing patients undergoing treatment protocol based on ICP+PbtO2 monitoring vs. ICP monitoring alone- found that a management protocol based on PbtO2 (target 20 mmHg) and ICP monitoring reduced the proportion of time with brain tissue hypoxia after severe TBI (0.45 in ICP-only group, 0.16 in ICP+PbtO2 group; *p* < 0.0001) and had a trend toward lower mortality and more favorable outcomes than ICP-only treatment. The ongoing BOOST-III study (ClinicalTrials.gov: NCT03754114) will aim to further clarify this issue.

Our results showed a higher threshold compared to the aforementioned literature, but were in agreement with Eriksson et al. (24), who found that the first 72 h of PbtO2<29 mmHg were associated with higher mortality. These results challenge the brain oxygenation threshold of 20 mmHg that has been used conventionally and delineates a time for monitoring PbtO2 that is predictive of outcome (5). However, we also found that hypoxic PbtO2 threshold changes with age. Recent evidence suggests that cerebrovascular autoregulation and ICP are profoundly dependent on age (25). For example, cerebral atrophy could buffer new pathological intracranial masses, which can be linked to a lower incidence of intracranial hypertension (26). Similarly, it is plausible that the need for higher target of PbtO2 in the elderly population could be due to a lower cerebrovascular reserve.

There are numerous factors that may affect PaO2 including pH, temperature and the oxygen saturation of Hb (27). Among these, CPP increase and consequent cerebral blood flow (CBF) optimization seem to be one of the major determinants (28).

Rosenthal et al. (27) explored whether PbtO2 more closely reflects variables related to cerebral oxygen diffusion or cerebral oxygen delivery and metabolism. In this study, patients underwent oxygen challenge (increase FiO2 100%), pressure challenge (increase arterial blood pressure) and carbon dioxide (CO2) challenge (hyperventilation). In multivariable analysis adjusting for various variables of cerebral oxygen delivery and metabolism, the only statistically significant relationship was that between PbtO2 and the product of CBF and cerebral arteriovenous oxygen tension difference (AVTO2), suggesting a strong association between brain tissue oxygen tension and diffusion of dissolved plasma oxygen across the blood-brain barrier. In our cohort, all hypoxic episodes had low CPP, although CPP < 60 did not prove to be an independent predictor of hypoxia. The prevalence of ICP elevation among hypoxic episodes (12/186, 6.1%) was comparable with general prevalence of ICP elevation over all episodes (18/303, 5.9%). 60.6% of the hypoxic episodes (PbtO2≤ 28) had ICP value lower than 20 and CPP value more than 60 mmHg, thus suggesting that normal values of ICP and CPP do not necessarily prevent hypoxia (22).

PbtO2 also depends on systemic oxygenation and ventilator parameters (10). Assuming normal alveolar function, PaO2 has a linear relationship with inspired oxygen (FiO2) (29). In patients with acute respiratory distress syndrome (ARDS), the PaO2/FiO2 ratio whilst on standard ventilator settings, is an appropriate tool to ensure correct categorization of patients with ARDS by disease severity (30). There is further evidence showing strong correlation between PbtO2 and FiO2 and a relationship between PbtO2 and PaO2 (31–33).

In these settings, FiO2 can be easily controlled, but PaO2 may be variable, therefore variations in PaO2 with FiO2 (as a result of the various dependent factors described above) will have an effect on the PbtO2, hence it will be dependent upon PaO2 fluctuations. As a result, the absolute PbtO2 value may be better interpreted whilst also considering the PaO2.

In the context of head injury, conventional physiology states that if CBF is reduced below a defined ischaemic threshold the affected areas of the brain must extract more oxygen from the limited blood supply to maintain cerebral metabolism (34). Through the use of oxygen-15 (15O) positron emission tomography (PET), authors show that the mean oxygen fraction (OEF) achieved significantly smaller OEF increases in hypoxic regions compared with normoxic regions, with similar falls in CBF (34). These findings imply that there may be other mechanisms contributing to tissue hypoxia that cannot be explained by classical physiological concepts relating to macrovascular brain ischemia (34). Menon et al. used an end-capillary oxygen tension (PvO2)—PbtO2 gradient in normoxic and hypoxic areas of brain tissue in patients with TBI to provide a measure of efficiency of microvascular oxygen delivery. The authors found that hypoxic areas of brain exhibited large diffusion gradients between tissue and end-capillary compared to normoxic regions, despite similar reductions in CBF (34). This can be explained by an increase in diffusion distance (increased tissue path lengths for oxygen) due to endothelial swelling, patchy microvascular collapse and peri-vascular edema. It is possible that based on these findings, PbtO2/PaO2 and PbtO2/FiO2 ratio may be a better indicator to severity of brain injury than absolute PbtO2.

The limitations of standalone PbtO2 monitoring was recognized by Arikan et al. (35) who were able to calculate a hypoxic threshold using a ratio of PbtO2/PaO2 suggesting that in the absence of hypoxemia (low PaO2) and at a constant cerebral metabolic rate of oxygen (CMRO2), ratios <0.10 indicated covert hypoxia and a deficient delivery of oxygen into the brain.

PbtO2/PaO2 ratio was applied to investigate the oxygenation status in tissue surrounding arterio-venous malformations (AVMs) and in the distant brain during surgery to better understand the perfusion pressure breakthrough phenomenon (36). In a cohort of 22 patients with supratentorial AVMs and 16 patients with cerebral aneurysm as controls, PbtO2/PaO2 ratio showed to be a better indicator than absolute PbtO2 in detecting intraoperative hypoxia in the margins of AVMs and in the distal ipsilateral brain. In our study, we found that PbtO2/PaO2<0.27 is an independent predictor of mortality. Our results confirm previous results but they also add new insight because of the higher number of patients and for the application of this index in traumatic brain injured patients and its correlation with outcome.

PbtO2/FiO2 ratio has never been studied so far, but- for the above mentioned reasons- it is plausible that it could be able to predict the severity of systemic and cerebral oxygenation impairment and could be potentially useful as part of the decision making for escalation of treatment after TBI (6). Finally, the PaO2/FiO2 threshold for mortality is consistent with the PaO2/FiO2 definition of ARDS and seems to be the most independent predictor of mortality comparing to other ratio, thus suggesting that the role of systemic oxygenation and mechanical ventilation is fundamental in brain injured patients (37–39).

### Limitations of the Study

The present study has several limitations that can interfere with the generalization of the results. First, this is a retrospective study and thus, some clinical data regarding the specific type of TBI injury and neurosurgical interventions were lacking. Second, the population studied is heterogeneous with different clinical presentations (as for GCS). Third, the study sample size is small both in terms of number of subjects and number of measurements per patient. Fourth, our dataset consisted of variable values at when the Pbto2 and Pao2 measurements were available. Conducting a prospective study while addressing these issues are needed for further validation of our findings.

## Conclusion

Among the determinants of cerebral oxygenation, systemic oxygenation seems to have the greater role. Therefore, appropriate mechanical ventilation to achieve appropriate oxygen targets is fundamental for the management of TBI patients. The critical threshold of PbtO2 could be related to patient's age; therefore, the threshold of cerebral oxygenation to consider treatment escalation should be made considering patients' specific and individualized need.

Indices such as the PbtO2/PaO2, PbtO2/FiO2, and PaO2/FiO2 ratios can be potentially used to discriminate patients at risk of mortality in TBI patients. We therefore believe that the inclusion of these indices may reflect the interrelationship between pathologies in the brain and lung in a more comprehensive fashion. The future work can include further validations of these findings in a prospective study.

## Data Availability Statement

The datasets generated for this study are available on request to the corresponding author.

## Ethics Statement

The study was approved by the Cambridge University Hospital Ethical Committee (30REC97/291) for anonymized data recording. The Ethics Committee waived the requirement of written informed consent for participation.

## Author Contributions

CR, AG, SA, RB, and PH contributed to the conception and design of the study. AG, CR, EB, MS, RB, and PH contributed to the acquisition of data for the work. SA and CR performed the statistical analysis with the collaboration of AG and PP. CR, SA, PP, and AG wrote the first draft of the manuscript. All authors revised and approved the final version of the manuscript.

## Conflict of Interest

The authors declare that the research was conducted in the absence of any commercial or financial relationships that could be construed as a potential conflict of interest.
